# Assessment of Chronic Sublethal Effects of Imidacloprid on Honey Bee Colony Health

**DOI:** 10.1371/journal.pone.0118748

**Published:** 2015-03-18

**Authors:** Galen P. Dively, Michael S. Embrey, Alaa Kamel, David J. Hawthorne, Jeffery S. Pettis

**Affiliations:** 1 Department of Entomology, University of Maryland, College Park, MD, United States of America; 2 Analytical Chemistry Branch, Biological and Economic Analysis Division, Office of Pesticide Programs, US EPA, Fort George G. Meade, MD, United States of America; 3 USDA-ARS Bee Research Laboratory, Beltsville, MD, United States of America; French National Institute for Agricultural Research (INRA), FRANCE

## Abstract

Here we present results of a three-year study to determine the fate of imidacloprid residues in hive matrices and to assess chronic sublethal effects on whole honey bee colonies fed supplemental pollen diet containing imidacloprid at 5, 20 and 100 μg/kg over multiple brood cycles. Various endpoints of colony performance and foraging behavior were measured during and after exposure, including winter survival. Imidacloprid residues became diluted or non-detectable within colonies due to the processing of beebread and honey and the rapid metabolism of the chemical. Imidacloprid exposure doses up to 100 μg/kg had no significant effects on foraging activity or other colony performance indicators during and shortly after exposure. Diseases and pest species did not affect colony health but infestations of *Varroa* mites were significantly higher in exposed colonies. Honey stores indicated that exposed colonies may have avoided the contaminated food. Imidacloprid dose effects was delayed later in the summer, when colonies exposed to 20 and 100 μg/kg experienced higher rates of queen failure and broodless periods, which led to weaker colonies going into the winter. Pooled over two years, winter survival of colonies averaged 85.7, 72.4, 61.2 and 59.2% in the control, 5, 20 and 100 μg/kg treatment groups, respectively. Analysis of colony survival data showed a significant dose effect, and all contrast tests comparing survival between control and treatment groups were significant, except for colonies exposed to 5 μg/kg. Given the weight of evidence, chronic exposure to imidacloprid at the higher range of field doses (20 to 100 μg/kg) in pollen of certain treated crops could cause negative impacts on honey bee colony health and reduced overwintering success, but the most likely encountered high range of field doses relevant for seed-treated crops (5 μg/kg) had negligible effects on colony health and are unlikely a sole cause of colony declines.

## Introduction

Honey bee (*Apis mellifera*) colony losses and declines in native pollinators have caused much concern worldwide [1–7]. In the United States, annual surveys conducted since the appearance of the syndrome known as colony collapse disorder (CCD) in 2006 continue to show consistent losses of colonies exceeding 30%, although the incidence of CCD has declined in recent years [8–10]. These losses threaten the economic viability of the beekeeping industry and have serious implications to pollination services for both cultivated and wild plants [11,12]. The consensus among bee scientists is that honey bee colony declines are the result of multiple stressors, working independently, in combination, or synergistically to impact honey bee health. Many stress factors have been identified, including parasitic mites (predominantly *Varroa destructor*), pathogens (viruses and *Nosema* spp.), interaction between mites and viruses, poor nutrition, pesticide exposure, management stress, and loss of foraging habitat [13–17]. While the specific causal pathways and relative contribution of these stressors are still unknown, beekeepers and many scientists assert that the extensive use of pesticides has had negative impacts on the health of honey bees and other pollinators.

Honey bees are exposed to pesticides used within the hive by beekeepers to control parasitic mites and pathogens, as well as to pesticides used to control pests and diseases of cultivated plants on which bees visit for nectar and pollen. Multiple studies conducted in Europe and the U.S. showed that both healthy and unhealthy colonies contained a diverse range of pesticides in pollen, honey, beewax, and bees [14,18–23]. In U.S. hive surveys, miticides (fluvalinate and coumaphos) used by beekeepers were the most frequently found, followed by pyrethroids, organophosphates, fungicides (mainly chlorothalonil), carbamates, and herbicides. In a recent study that collected pollen from bee hives in seven major crops, 35 different pesticides were detected with a total residue load ranging from 23.6 to 51,310 μg/kg from an average of 9.1 pesticides per pollen sample [24]. Honey bees have probably been exposed to these pesticide loads for many years prior to 2006, yet there has been no evidence linking hive residues of an individual chemical or combination of chemicals to recent honey bee declines, particularly the rapid colony depopulation that is characteristic of CCD [14,16,19]. Some studies have actually shown that residue levels of coumaphos and the pyrethroid esfenvalerate were lower in CCD-affected colonies [14], and expression of genes involved in pesticide detoxification in collapsed colonies was not different compared to control colonies [25].

Despite the lack of evidence implicating pesticides as a major causal factor, neonicotinoid insecticides have been widely implicated in adversely affecting honey bee health due to their extensive use worldwide, systemic activity, and presence in pollen and nectar. These insecticides are very effective on a broad spectrum of insect pests [26] but also moderately to highly toxic to honey bees depending on the particular active ingredient. Six neonicotinoids, including imidacloprid, thiamethoxam, clothianidin, acetamiprid, thiacloprid, and dinotefuran, are applied as systemics on many crops that require managed honey bee colonies and non-*Apis* bees to attain economic yields. Neonicotinoids bind agonistically to the post-synaptic nicotinic acetylcholine receptors in the insect central nervous system, causing spontaneous discharge of nerve impulses and eventual failure of the neuron to propagate any signal [27–29]. In this study, we focused on imidacloprid because it is the most widely used and has drawn more attention to bee health issues than other neonicotinoids. As the first neonicotinoid registered in the U.S. in 1994, imidacloprid is now generic and currently used in over 400 products, accounting for about one-fifth of the global insecticide market [30]. Imidacloprid is also used on home gardens, turf, ornamental shrubs and trees at application rates much higher than label rates for agricultural crops. For many labeled uses on agricultural crops, imidacloprid can be applied at planting as seed coatings or soil treatments but also by chemigation, side-dress treatment, or foliar spray during the crop cycle (including during flowering if bees are not actively foraging) [31]. Due to their systemic activity, imidacloprid and other neonicotinoids are absorbed by the roots or leaves and then xylem transported in the vascular system through the plant, where they may persist for weeks or months following application depending on the application rate and abiotic conditions. Generally, these chemicals are less likely to move translaterally from leaves to the fruiting structures; however, there is increasing evidence that they move to some extent into pollen and nectar [32–35]. For these reasons, many reports claim that neonicotinoids, particularly imidacloprid, are the major causal factor affecting honey bee health and also may act to trigger other stresses on bees. However, there is no scientific evidence to link neonicotinoids as the major cause of colony declines [36]. A recent workshop of bee experts evaluated the relationship of 39 candidate causes to colony declines and judged neonicotinoids to be a possible contributing factor but unlikely the sole cause [37].

Several review papers [38–40] present comprehensive accounts of the available data on residue levels and exposure risks of neonicotinoids and other pesticides to bees. Of the exposure routes outside the hive, residue studies have detected imidacloprid at average levels of 2–3.9 μg/kg in pollen and less than 2 μg/kg in nectar of seed-treated corn, sunflowers and rape [41–43]. More recent studies of treated cucurbit crops revealed higher residues of imidacloprid and other neonicotinoids in field-collected pollen and nectar, particularly when insecticides were applied at higher rates and closer to flowering [34,35]. Residue levels of imidacloprid ranged 24 to 101μg/kg in pollen and 7 to 16 μg/kg in nectar in pumpkin plants receiving the high label rate, delivered as a transplant water application and later by drip irrigation during bloom [35]. Other routes of exposure to foraging bees include residues of neonicotinoids in surface water, guttation droplets exuded from treated corn seedlings, and contaminated talc dust from planter exhaust [44–47]. Of the imidacloprid concentrations detected inside the hive, residues in bees, bee bread and other hive matrices have been consistently lower than residues in pollen collected directly from flowers and also lower and much less frequent than other pesticides. The highest levels in pollen collected at the hive entrance have been reported in France, where imidacloprid residues were detected in 40.5% of the samples, with levels ranging from 0.9 to 3.1 μg/kg [48,49]. In these studies, levels were either below the limit of detection or <2 μg/kg. Other studies in Europe reported lower detection frequencies in bee-related matrices [50], and less than 3% of the pollen samples collected from U.S. colonies contained neonicotinoid residues, of which most were the less toxic acetamiprid and thiacloprid [21]. The reported concentrations of imidacloprid in pollen and nectar from seed-treated crops (<5 μg/kg) are not acutely lethal to honey bees based on dietary LD_50_ values. However, these residues are near to levels shown to cause sublethal effects in laboratory studies [38]. In general, published results suggest that exposure levels above 20 μg/kg of imidacloprid can lead to subtle physiological and behavioral abnormalities in honey bees, including reductions in associative learning, queen fecundity and foraging activity, as well as increased susceptibility to other stresses [38,51–54]. For example, some studies have shown interactions of sublethal doses of imidacloprid with other chemicals [55] and with the gut parasite *Nosema* [56], resulting in increased susceptibility to pesticides and increased disease infection levels, respectively. While there are demonstrated side effects on honey bees from sublethal doses of imidacloprid, laboratory results are conflicting and some disagreed with the no effects observed in field studies [37]. For example, chronic exposure of honey bee hives for 39 days to field realistic concentrations of imidacloprid (2–20 μg/kg) in sunflower nectar did not result in increased worker mortality or overwintering loss [39,57]. Furthermore, most laboratory studies measured sublethal effects on individual bees (or larvae) or small cohorts of workers by exposing them orally or topically to single doses of pesticides in sucrose solution or contaminated pollen. These effects are most likely less disruptive to the overall health of a functional colony than the direct effects on individual bees. The honey bee colony, as a superorganism, can compensate for many stress factors as a result of the social interactions and feedback mechanisms between individual bees. A meta-analysis reported that the results of impaired learning effects from neonicotinoids exposed to individual bees in laboratory tests cannot be extrapolated to a real exposure scenario under field conditions [58]. Thus, chronic lethal and sublethal effects of pesticide exposure need to be assessed at the colony level.

Few field studies using honey bee colonies have examined sublethal effects of dietary exposure to imidacloprid or other neonicotinoids over multiple brood cycles. One study fed replicate colonies with repeated imidacloprid doses of 0.5 and 5 μg/kg in sucrose syrup and showed no immediate or delayed mortality effects [57]. Several long-term studies found no detrimental effects on colonies placed during flowering in fields of clothianidin-seed treated and untreated canola [33], in oilseed rape fields treated with foliar-applied and seed-treated neonicotinoids [59], and in tunnel cages enclosed over thiamethoxam-seed treated oilseed rape and maize [60]. A similar study investigated the potential impact on honey bee mortality in 16 apiaries surrounded by variable land use of imidacloprid seed-treated corn fields [50]. They reported a negative correlation between colony mortality rate and the acreage of treated fields, suggesting imidacloprid had no adverse effect on colony health. Two whole colony experiments involving sublethal exposure of neonicotinoids to honey bees showed adverse impacts on forager longevity, homing activity, and winter survival of colonies, but these studies used unrealistically high field doses and routes of exposure [61,62]. More recently, Sandrock et al. [63] reported that sublethal dietary exposure to field relevant concentrations of thiamethoxam and clothianidin had significant negative short-term and long-term effects on colony performance and queen health.

To date, no field study has shown that imidacloprid adversely affects honey bee colony health when directly exposed to field realistic dietary doses. Here we present results of a study examining the chronic sublethal effects on whole honey bee colonies fed supplemental pollen diet containing imidacloprid at field realistic doses for 12 weeks. Various endpoints of colony performance and foraging behavior were measured during and after exposure, including winter survival. We also present data from a related experiment that addressed the fate of imidacloprid within colonies and the quantification of the actual exposure dose to worker bees, brood and the queen via honey, beebread and royal jelly.

## Methods

### Sublethal Experiments

#### Test colonies

We conducted separate experiments using new colonies each year during 2009–10 to assess the chronic sublethal effects of prolonged exposure to imidacloprid. Colonies were hived with starter pIcHive ackages of 900 g of bees obtained from a commercial supplier (Wilbanks Apiaries; Claxton, GA, USA) during early April. Laying sister queens originating from the same breeding line were used to ensure uniform genetic makeup of bees among treatment groups. In each year, new hive boxes with 10 frames and plastic foundation were used to eliminate possible carryover contamination. Colonies were located on the University of Maryland research farm at Beltsville, MD in areas relatively free from insecticide exposure. Crops within the foraging range of about 3 km of the apiaries were exclusively field corn, soybean, and small grains. None of these crops were treated with imidacloprid, although a portion of the corn acreage was seed-treated at the low rate with other neonicotinoids. For the first four weeks, colonies were fed sucrose syrup to allow colonies to build up before they were assigned to treatment groups. During this period, several inspections were conducted to check queen status and colony development. Queens were replaced if colonies became queenless or showed signs of a weak egg-laying queen. During early May, a detailed inspection of colonies was conducted to record bee strength and brood production, at which time brood frames with workers were exchanged among hives to equalize colony strength. Colonies were then randomly assigned to treatment groups and relocated to isolated apiaries on the research farm. Individual hives were placed on wood platforms spaced 10 m apart in each apiary.

#### Treatment regimes

In each year, colonies were assigned to four treatment groups: no exposure (control), 5, 20 and 100 μg/kg of imidacloprid. The 5 and 20 μg/kg doses represented the reported high range of residues present in pollen and nectar in seed-treated crops. The 100 μg/kg dose was considered a worse-case exposure resulting from imidacloprid treatments applied to crops during bloom. Residues of neonicotinoids in pollen of pumpkin plants treated with labelled rates of imidacloprid can actually reach 100 μg/kg [35]. The experimental design in 2009 and 2010 included ten and seven replicate colonies of each treatment, respectively. Each of five apiaries in 2009 included two replicates of each treatment, while one replicate set of treatments was located at seven apiaries in 2010.

#### Exposure method

After colonies were assigned treatment groups, they were allowed to freely forage but each colony was provisioned with a pollen diet substitute (MegaBee, Dadant & Sons, Inc., Hamilton, IL) either untreated or spiked with imidacloprid. Stock solutions of imidacloprid were prepared from the formulated product [Admire Pro (42.8% a.i.), Bayer CropScience, Raleigh, NC] and diluted in distilled water. The final concentration of each treatment dose was obtained by adding the appropriate concentrated solution of imidacloprid to heavy sucrose syrup (2 to 1 parts of sugar and water), which was then added to the MegaBee powder in a 1.7:1 diet to sucrose solution ratio. This produced soft, moist dough which was formed into 80 g patties. Four diet patties were placed weekly on the top bars of frames inside each colony to allow bees *ad libitum* access to the pollen substitute. At each diet placement, we removed and weighed remaining portions of the old patties to keep track of the cumulative weight of diet consumed per colony. Pollen traps were installed at the entrance of each hive to induce bees to consume maximum amounts of the diet. In both years, treatment regimes began in mid May and continued for 12 weeks, ending in early August. This exposure method represented a worse-case scenario of exposure to residues entering colonies in pollen collected from multiple bloom events of treated crops. Imidacloprid doses were either directly or indirectly exposed to life stages for at least two or three brood cycles. To verify the exposure dose, samples of fresh patties of each treatment dose and portions of patties removed after 7 days were collected and analyzed for imidacloprid residues. To confirm exposure within colonies, samples of hive bees and bee bread were also collected several times after the exposure period and analyzed for residues.

#### Measured endpoints

Colonies were sampled biweekly to estimate the percentage of the frame area covered with drawn cells, bees, capped brood, cells with older larvae, and cells packed with beebread and honey. Each frame was carefully removed and held above the hive box to visually estimate the percentage of area covered by each endpoint on each side. Endpoints were recorded concurrently on each replicate set of treatment colonies within an apiary by pairs of inspectors who voiced data to recorders. To minimize estimation bias, inspectors rotated evenly among the different treatment colonies within each replicate set. The presence and egg laying status of the queen was established during each inspection, either by directly observing her or freshly-laid eggs. Additional notes were recorded on any unusual presence of drone cells, dead larvae, abnormal behavior of workers, abnormal brood pattern, and signs of disease or pest presence. To measure foraging activity, we recorded data twice weekly on the weight of pollen collected in the entrance traps, and data biweekly on the number of foraging bees returning with and without pollen loads. Foraging counts at the hive entrance were tallied over a 5-minute period in the morning between the hours of 9 a.m. and 11 a.m.

#### Hive manipulations

Queen cells were removed from combs during inspections to prevent swarming. During the first half of the exposure period, missing or weak queens in all treatment groups were replaced to minimize breaks in brood rearing. However, there was no manual queen replacement thereafter and colonies were left to replace queens naturally. In both years, a second full box was added in mid-May and a super in mid-June to each colony to prevent overcrowding and the swarming instinct. After the treatment exposure ended in early August, pollen traps were removed and colonies were then fed heavy sucrose syrup from mid August through the fall using top feeder pails to build up honey stores in preparation for overwintering. Based on local beekeeping recommendations, each colony was given sucrose syrup until approximately 30 kg of honey was stored or until the bees stopped feeding. In both years, a detailed assessment of colony health and performance was made in early October to estimate bee strength, brood development, food stores, and queen status. Additional inspections were conducted in January, February and March to assess food stores and overwintering survival.

#### Disease determinations

In 2009, hive bees from each surviving colony were collected in early November and again the following May for disease diagnosis. Samples containing approximately 100 bees were randomly removed from brood frames, placed in 70% alcohol, and submitted to the USDA-ARS Bee Research Laboratory at Beltsville, MD, where they were examined for *Varroa* mites, tracheal mites, and *Nosema* disease. Similarly, samples were collected at the end of the 2010 exposure period and examined for both mites and *Nosema* disease.

### Within-colony Fate Experiment

An additional experiment was conducted in 2011 to track the movement and degradation of imidacloprid within whole colonies to better understand the fate of imidacloprid doses used in the sublethal experiments. In early April, we established 24 colonies in new hive boxes (8 foundation and 2 drawn frames) with 900 g packages of bees and sister queens obtained from the same commercial supplier mentioned above. Bees were fed sucrose syrup and MegaBee diet patties for five weeks to build up colonies before they were assigned to treatment groups. During mid May, hives were inspected to assess and equalize bee and brood densities and a second full depth box with foundation frames was added to allow expansion. Colonies were then assigned to three treatment groups (each with 8 replicates) and relocated to four isolated apiaries, each with two replicates of treatments. All colonies were fed 2000 g of sucrose syrup (SS) and 400 g of diet patties (DP) provisioned each week for six weeks. The control group was exposed to untreated SS and DP. A second group was exposed to 20 μg/kg imidacloprid in SS and untreated DP to mimic the high range of residue exposure via contaminated nectar. The third group was exposed to 100 μg/kg in DP and untreated SS to mimic the high range of residue exposure via contaminated pollen. The spiked 2000 g of SS contained the same amount of imidacloprid as the spiked 400 g of DP, so all treated colonies were exposed to 40 μg of the active ingredient each week by both routes of exposure.

At 2, 4 and 6 weeks during exposure and again at 6 weeks after exposure, we collected samples of bees, bee bread, honey, and larvae to measure residues of imidacloprid. To avoid cross-contamination, separate collection tools were assigned to each treatment group of colonies. Samples of 30–40 hive bees were removed by gently scooping clusters of bees from brood frames using 250 ml snap-seal plastic containers. Older larvae (approx. 30–40) were removed individually from brood cells using tweezers. A putty knife was used to remove a section (approx. 9 cm^2^) of comb with cells packed with bee bread, which was carefully separated from the wax cells using tweezers and probes. Honey was removed with plastic containers to scrape up through capped cells, allowing honey to ooze out. Additionally, we removed all queens after five weeks of exposure to trigger queen cell formation and production of royal jelly. Five days later, grafting tools were used to scoop out royal jelly from queen cells. Queens removed were banked in other colonies until enough royal jelly was collected and then returned to their respective hives. All samples in the field were stored on ice in coolers and then frozen immediately to -80 C., after which they were further processed in the laboratory. For most samples, we collected quantities greater than 3 g in order for the chemical analysis to measure residues at the lowest detection level; however, there were some samples of larvae that were less than 3 g.

Possible treatment effects on queen health and colony performance were also monitored every two weeks during the entire experiment. A third hive box was added after exposure to allow space for further colony expansion. Full inspections of frames to record endpoints of bee strength, brood and food stores were made after 4 and 6 weeks of exposure according to the methods described above. Less quantitative inspections were conducted at 2, 4 and 6 weeks after exposure by recording the number of inner spaces between frames with clusters of bees, number of frame sides with stored honey and beebread, and the number of frame sides covered with at least 25% capped brood and larval cells. Colonies were fed sucrose syrup for several weeks starting on late August, and then a final inspection was made on 24 September to assess the cumulative impact of the treatments. Colonies were rated ‘weak’ (missing queen, very little brood and stored food), ‘medium strength’ (queen and brood but low in stored food), and ‘strong’ (queen, brood and sufficient stores of food present).

### Residue Analysis

Samples of bees and other hive matrices from all experiments were analyzed for residue levels of imidacloprid and its major metabolites (imidacloprid olefin, 5-hydroxy imidacloprid, 6-chloronicotinic acid, imidacloprid urea, desnitro imidacloprid olefin, and desnitro imidacloprid HCl). All samples were processed by the Analytical Chemistry Branch, Biological and Economic Analysis Division, Office of Pesticide Programs, U.S. EPA at Fort George G. Meade. Sample preparation, calibration standards, and residue analysis using liquid chromatography−tandem mass spectrometry (LC-MS/MS) were preformed according to the method protocol described in [64,35]. The limit of detection was 0.2 for imidacloprid and 0.2–15 μg/kg for the metabolites.

### Statistical Analysis

Estimates of individual frame area covered by each measured endpoint at each inspection were weighed according to the size of the hive box (i.e. lower weight assigned to super frames) and then averaged over all frames per colony. For bee strength, a linear regression function was used to estimate the number of hive bees per colony from the percentage frame area covered with bees [65]. Exact estimates of bee strength were not possible because colonies were inspected during the day when foragers away from the hive were not included. A mixed model ANOVA procedure (SAS Institute, version 9.1.3) was used to test for treatment effects on colony performance and foraging. Each colony represented a single experimental unit and apiaries were treated as a random blocking factor. All data sets were evaluated before analysis for normality and homogeneity of variances by examining residual plots and Shapiro-Wilk statistic. For data not meeting the assumptions of ANOVA, the arcsine transformation for percentages or other appropriate transformations were used or variances were grouped among subsets of treatment groups prior to analysis. For endpoints measured over time, inspection date was treated as a fixed factor and the repeated measures option was used to correct for autocorrelation among inspections. Means were separated following a significant *F* test by using Tukey’s multiple comparison adjustment (*p* < 0.05). For endpoints with non-significant interaction effects, contrasts and adjusted *p* values were computed comparing the overall treatment effect with the control.

Additional tests were performed to examine the dose effect on endpoints of overall colony performance using linear regression. Here, we converted the data collected for each endpoint over inspection dates to cumulative area under the curve (AUC) values. Area at each inspection was derived by averaging the endpoint measurement over two sequential inspection dates and multiplying by the number of days between dates. Area values were then accumulated over the entire exposure and post exposure period. Regression analysis fitted a linear model with exposure dose to the total area value of each colony endpoint. We also used Fisher’s Exact Test to determine if the frequency of queen events and overwintering survival were significantly related to the exposure dose. To increase statistical power, we combined data over both years on the number of surviving colonies at the October, February and March inspection dates. We used the GLIMMIX procedure of SAS to fit a logistic regression model with exposure dose to the proportion surviving over the three dates. The analysis accounted for a random year effect and adjusted for autocorrelation among months within each year. Contrast tests with Tukey’s multiple comparison adjustment (*p* < 0.05) determined if overall survival of the exposed colonies at each treatment dose was significantly different from survival of the control colonies. For the residue analysis data, levels of the parent and metabolite compounds in bees, hive matrices, and diet patties were averaged to calculate means and ranges for each treatment dose. Non-detected samples were scored a value of zero for calculating means or in some cases means were computed using only positive detections to show upper range exposure levels. Regression analysis and Spearman correlations tested the degree of association between disease occurrence in colonies (incident and severity of mites and *Nosema* spores) and dose of imidacloprid exposure.

## Results

### 2009 Sublethal Experiment

#### Imidacloprid exposure

Exposure doses were confirmed by residue levels in partially-consumed patties removed after 7 days, which averaged 0.0, 5.5, 19.8 and 97.5 μg/kg of imidacloprid from the control, 5, 20 and 100 μg/kg colonies, respectively. Weekly consumption of diet patties varied significantly over the exposure period (*F*_(11, 428)_ = 4.31, *p* <0.001) but was not different among treatment groups, which ranged from 265.3 to 277.2 g per colony. Based on total consumption over 12 weeks, each colony of the 5, 20 and 100 μg/kg treatment groups was exposed to an average cumulative dose of 16.6, 63.7 and 322.6 mg of imidacloprid, respectively. Imidacloprid and several metabolites were present in 40% of the bee samples and 68% of the bee bread collected from colonies in August (end of the exposure period). Of the positive detections, residues ranged from 0.3 to 2.8 μg/kg in bees and 0.4 to 1.6 μg/kg in beebread, and were generally higher with increasing exposure dose. Only traces of residues (<0.4 μg/kg) were detected in 24% of the bee samples collected in October, whereas residues in 85% of the bee bread samples were consistently higher than levels in August but not correlated with exposure dose (*r* = 0.282; *p* = 0.145). Less than 5% of the control samples had trace amounts of imidacloprid. Bee bread residues of the positive samples averaged 0.7, 1.2, 2.8 and 2.2 μg/kg in bee bread from the control, 5, 20 and 100 μg/kg treatment groups, respectively.

#### Colony health

No colonies showed any evidence of overcrowding, unusual queen cell formation, or swarming behavior during or after the exposure period. The number of queen cells of all types summed over inspections was not significantly affected by the treatments (*F*_(3, 31)_ = 0.87, *p* = 0.47). Eleven queen events occurred at different times among the 40 colonies, and the frequency of events was positively associated with the exposure dose (*p* = 0.009, Fisher’s Exact Test). Of these events, a weak queen in one control colony was replaced manually in late June, while supersedural events occurred during late summer in 2, 4 and 4 colonies exposed to 5, 20 and 100 μg/kg doses, respectively. All colonies survived to enter overwintering, except for two colonies in each higher dose groups, which became too weak after queens were naturally replaced and thus had to be terminated in early September to avoid robbing.

All colonies sampled in the fall were infested with *Varroa* mites at average (±SE) densities of 7.1 ±1.4, 8.8 ±2.6, 6.6 ±1.2 and 13.3 ±3.0 mites per 100 bees from the control, 5, 20 and 100 μg/kg treatment groups, respectively. Mite counts in colonies exposed to the high dose were significantly higher than the control group based on a contrast test of the difference (*F*_(1, 28.4)_ = 4.31, *p* = 0.047). Furthermore, *Varroa* infestations showed a positive linear relationship with imidacloprid dose (*p* = 0.03). Only three colonies (two exposed to 5 μg/kg; one exposed to 20 μg/kg) in the fall tested positive for *Nosema* spores (< 1.5M spores per bee). Of the colonies that survived to the following spring, *Varroa* densities ranged from 6.7 to 9.0 mites per 100 bees and were not significantly different among treatment groups. Eleven of the 27 colonies that survived the winter were infected with *Nosema* spores (< 2.2M spores per bee) but spore counts were not positively associated with exposure doses. Dead bees and queens from seven colonies that died during the winter were also analyzed for *Nosema* spores and only one colony in each 5 and 20 μg/kg exposed groups tested positive.

**Fig 1:**
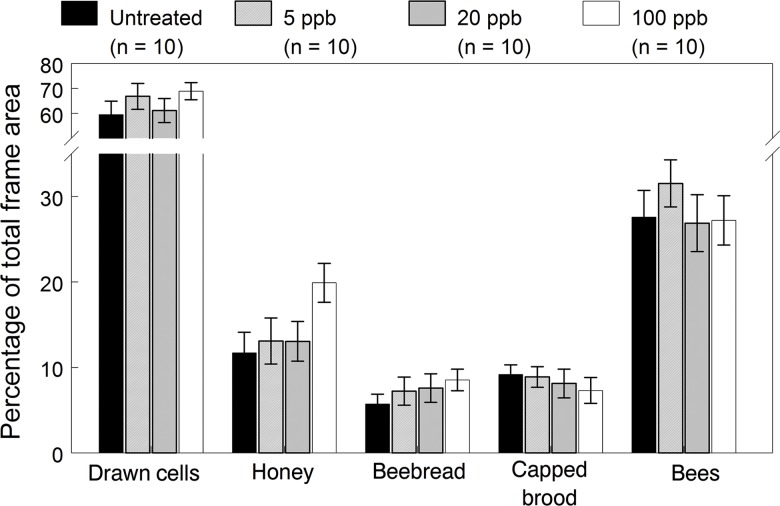
Colony performance endpoints of the 2009 colonies exposed for 12 weeks to untreated or imidacloprid-spiked diet patties. Means (±SE) are given for each endpoint recorded at the end of exposure (August 6).

**Fig 2:**
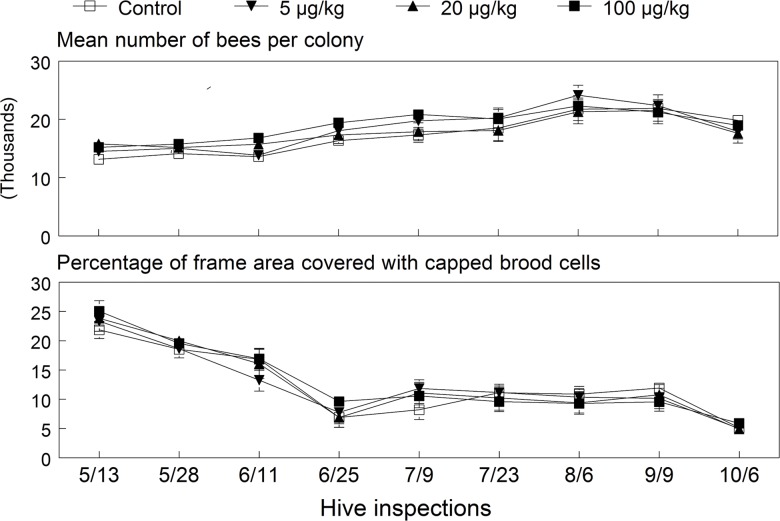
Mean (±SE) colony size and percentage of capped brood cells in the 2009 colonies exposed to untreated or imidacloprid-spiked diet patties for 12 weeks (May 15 to August 8). ANOVA results for bees (dose: *F*_(3, 26.7)_ = 0.99, *p* = 0.414; date: *F*_(8, 281)_ = 75.25, *p* < 0.001; interaction: *F*_(24, 281)_ = 1.13, *p* = 0.311) and brood (dose: *F*_(3, 25.9)_ = 0.06, *p* = 0.980; date: *F*_(8, 279)_ = 68.27, *p* < 0.001; interaction: *F*_(24, 279)_ = 0.69, *p* = 0.859).

Colony performance endpoints of bees, capped brood cells, food stores and drawn out cells showed no significant dose effects at the end of the exposure period (S1 Table, Fig. 1). The only difference observed was the consistently higher levels of honey stores in exposed colonies compared to the control (contrast test: *F*_(1, 31.1)_ = 4.63, *p* = 0.039). This effect was also supported by a significant linear positive relationship between the cumulative AUC values for capped honey and imidacloprid dose (*p* = 0.02). Fig. 2 shows the mean bee colony size and percentage of frame area with capped brood over all inspection dates for each exposure group of surviving colonies. These endpoints varied significantly over inspection dates but were not significantly affected by dose or its interaction with date. The colony size (±SE) averaged over all dates was 17,440 ± 546, 18,541 ± 565, 17,813 ± 540 and 18,850 ± 448 bees in the control, 5, 20 and 100 μg/kg treatment groups, respectively. However, as mentioned above, two colonies in each of the higher dose groups were terminated in early September; thus, means of the last two inspection dates for these groups are based on the eight surviving colonies. Interestingly, regression results showed a near significant dose relationship (*p* = 0.054) with cumulative AUC values of bee numbers, indicating higher overall colony size with higher exposure doses. Dose relationships using AUC values were not significant for capped brood cells and older larvae (*p* = 0.54), pollen (*p* = 0.75), queen cells (*p* = 0.19), and drawn cells (*p* = 0.13).

**Fig 3:**
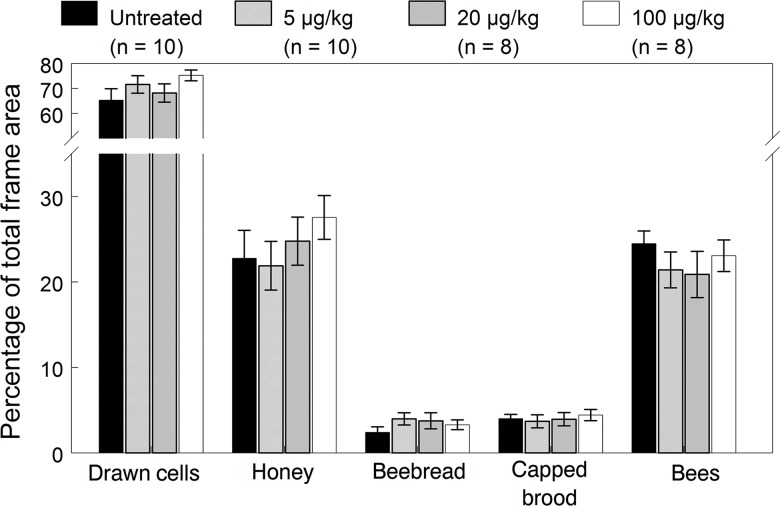
Colony performance endpoints (mean±SE) of the 2009 colonies recorded on October 6 prior to overwintering (two months after exposure).

Of the remaining colonies surviving on October 6, we found no significant differences in the cells drawn, capped honey, bee bread, capped brood and bees (Fig. 3, S2 Table), although honey stores in the 20 and 100 μg/kg exposed colonies were consistently higher at the August and October inspections. Despite similar performance endpoints among treatments prior to overwintering, the higher rates of queen events and resultant breaks in brood rearing at the higher doses apparently weaken colonies during the winter, resulting in lower survival. We considered colonies to have successfully overwintered if they survived to the March inspection with an active queen with brood and were able to buildup in the spring. Out of ten replicate colonies in each treatment group, 10, 8, 7 and 6 colonies survived the winter from the control, 5, 20 and 100 μg/kg groups, respectively. Hive inspections of die-off colonies showed smaller clusters that either could not reach the stored honey or were over-chilled but there were no food shortages or symptoms of colony collapse disorder related to winter mortality. Using Fisher’s one-tailed Exact Test, only the difference in winter survival between the control and 100 μg/kg exposed colonies was statistically significant (*p* = 0.043).

#### Foraging measurements

**Fig 4:**
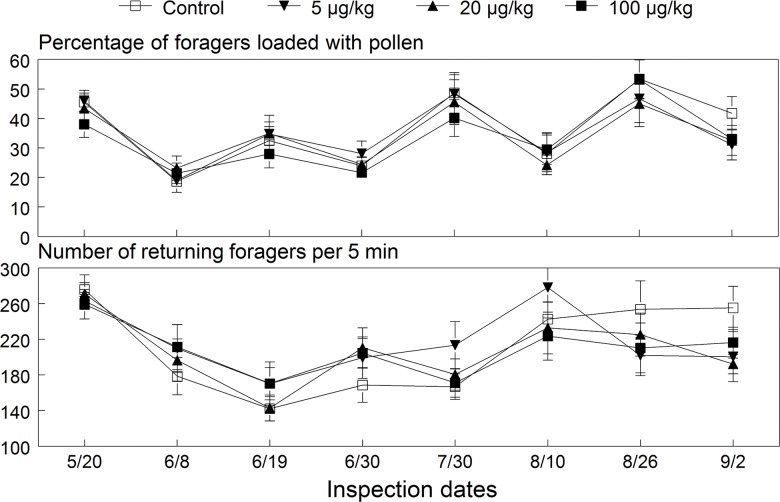
Mean (±SE) percentage of foragers with pollen pellets and the number of foragers returning to the 2009 colonies exposed to untreated or imidacloprid-spiked diet patties. ANOVA results for the percentage loaded with pollen (dose: *F*_(3, 93.6)_ = 0.53, *p* = 0.662; date: *F*_(7, 176)_ = 17.81, *p* < 0.001; interaction: *F*_(21, 177)_ = 0.39, *p* = 0.993) and number of foragers (dose: *F*_(3, 86.8)_ = 0.43, *p* = 0.733; date: *F*_(7, 177)_ = 11.78, *p* < 0.001; interaction: *F*_(21, 178)_ = 1.02, *p* = 0.445).

The amount of pollen collected twice weekly in the entrance traps showed a significant time effect (*F*_(20, 690)_ = 28.04, *p* < 0.001) but no dose (*p* = 0.37) or interaction (*p* = 0.99) effects on foraging success. Pollen collected by the control, 5, 20 and 100 μg/kg exposed colonies averaged 56.6 ± 3.0, 60.8 ± 2.9, 57.6 ± 2.6 and 59.8 ± 2.3 g (±SE) per day, respectively. No effect was further indicated by a non-significant regression relationship between the cumulative AUC values for collected pollen and imidacloprid dose (*p* = 0.81). We also found no significant main dose or interaction effects on the number of foragers returning to each hive, which ranged from 206 to 217 bees per 5 minutes; and no dose and interaction effects on the percentage of bees loaded with pollen pellets (Fig. 4). However, foraging activity of the exposed colonies was significantly 12% lower than that of control hives during August and September (contrast test: *F*_(1, 28.7)_ = 5.50, *p* = 0.026).

### 2010 Sublethal Experiment

#### Imidacloprid exposure

The second year experiment followed the same protocol used in 2009, except for only seven replicates per treatment and that the 12 week exposure ended later in August. Individual colonies in treatment groups consumed similar amounts of diet patties, ranging from an average of 58.8 to 61.7 g per day. Exposure doses were confirmed by residues in both old and fresh treated patties, all of which were within ± 5% of the targeted concentrations of 5, 20 and 100 μg/kg. Imidacloprid presence in the control colonies was non-detectable, while residues in bees and beebread collected from exposed colonies were less frequently detected and lower than levels found in 2009. Residues of 0.3–2.2 μg/kg in bees were detected after four weeks of exposure in two colonies exposed to 100 μg/kg, while only trace amounts of imidacloprid (< 0.5 μg/kg) in bees were detected in three of the 28 colonies sampled on August 19. Beebread samples collected from all colonies on June 30, August 16 and October 7 showed imidacloprid residues ranging from 0.2 to 4.1 μg/kg in only 4 or 5 exposed colonies on each date. These residues consistently decreased after exposure and were not related to exposure dose.

#### Colony health

Similar to the 2009 experiment, colonies expanded in size without any signs of swarming by utilizing space in hive boxes added during the early summer. However, unlike the 2009 results, the frequency of queen events was not associated with exposure dose (*p* = 0.83, Fisher’s Exact Test), nor was there a significant regression relationship between the number of supersedural cells and dose (*p* = 0.70). Control and 5 μg/kg exposed colonies actually experienced nine queen events compared to five events in colonies exposed to the higher doses. All colonies sampled on August 19 were infested with *Varroa* mites at average densities (±SE) of 2.0±0.39, 1.8±0.56, 2.9±0.99 and 3.9±0.82 mites per 100 bees from the control, 5, 20 and 100 μg/kg treatment groups, respectively. Although mite counts were not significantly different among treatment groups (*F*_(3, 23)_ = 2.01, *p* = 0.14), regression results showed a significant increasing trend with exposure dose (*p* = 0.043). Only one control and two treated colonies tested positive for low levels of *Nosema* spores.

**Fig 5:**
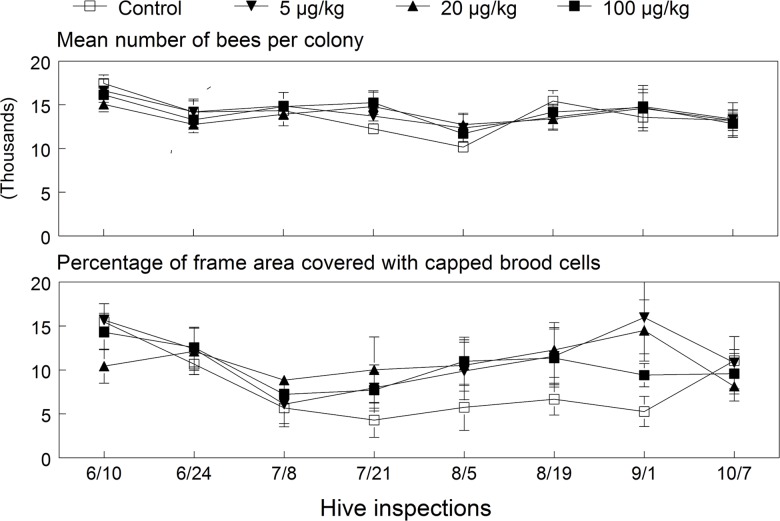
Mean (±SE) colony size and percentage of capped brood cells in the 2010 colonies exposed to untreated or imidacloprid-spiked diet patties for 12 weeks (May 27 to August 19). ANOVA results for bees (dose: *F*_(3, 23.7)_ = 0.04, *p* = 0.988; date: *F*_(7, 165)_ = 7.69, *p* < 0.001; interaction: *F*_(21, 165)_ = 3.89, *p* = 0.608) and brood (dose: *F*_(3, 18.3)_ = 0.34, *p* = 0.800; date: *F*_(7, 146)_ = 8.64, *p* < 0.001; interaction: *F*_(21, 146)_ = 1.16, *p* = 0.296).

**Fig 6:**
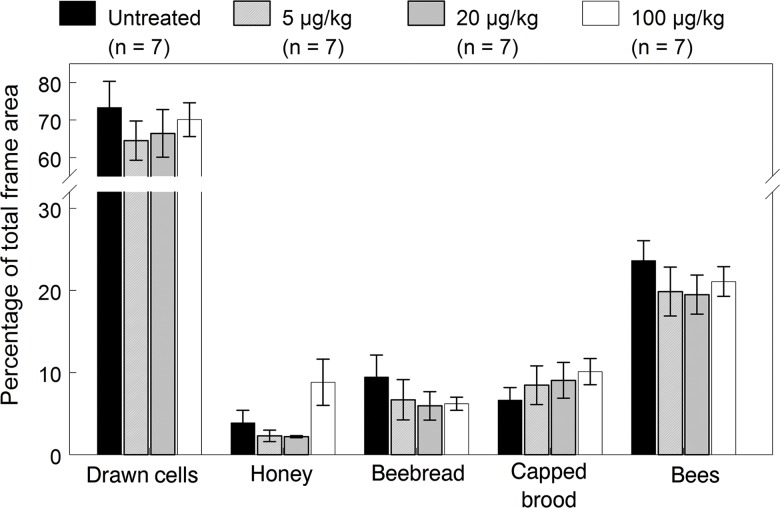
Colony performance endpoints of the 2010 colonies exposed for 12 weeks to untreated or imidacloprid-spiked diet patties. Means (±SE) are given for each endpoint recorded at the end of exposure (August 19).

The process of hiving packages of bees started later in April and cooler temperatures slowed the build-up of colonies; thus, bee populations were overall lower than those in 2009. Colony population estimates (±SE) across all inspection dates averaged 13,822±600, 14,200±790, 13,813±690 and 14,140±613 bees in the control, 5, 20 and 100 μg/kg treatment groups, respectively. The number of bees per colony and percentage of frame area covered with brood (capped cells and older larvae) varied over inspection dates but were not significantly affected by the exposure dose (Fig. 5). Brood production significantly changed over the season, with expected higher levels in all colonies during June, lowest during July, and then a gradual increase through to September. Note that the brood was lowest in control colonies during late July through early September due to a higher frequency of queen events and subsequent breaks in reproduction. Although the dose and interaction effects were not significant, a contrast test showed a significant difference in brood production between the control group and exposed colonies grouped together (*F*_(1,186)_ = 6.22, *p* = 0.014). An analysis of hive inspection data on August 19 after exposure ended revealed no significant dose effects on any of the colony performance endpoints (Fig. 6, S3 Table). Although the 100 μg/kg exposed colonies stored higher amounts of honey on August 19, consistent with results in 2009, the relationship between the honey stores based on AUC values accumulated over all dates and exposure dose was not significant (*p* = 0.98). Dose relationships using AUC values were also not significant for bee numbers (*p* = 0.92), capped brood cells and older larvae (*p* = 0.85), pollen (*p* = 0.25), queen cells (*p* = 0.70), and drawn cells (*p* = 0.92).

**Fig 7:**
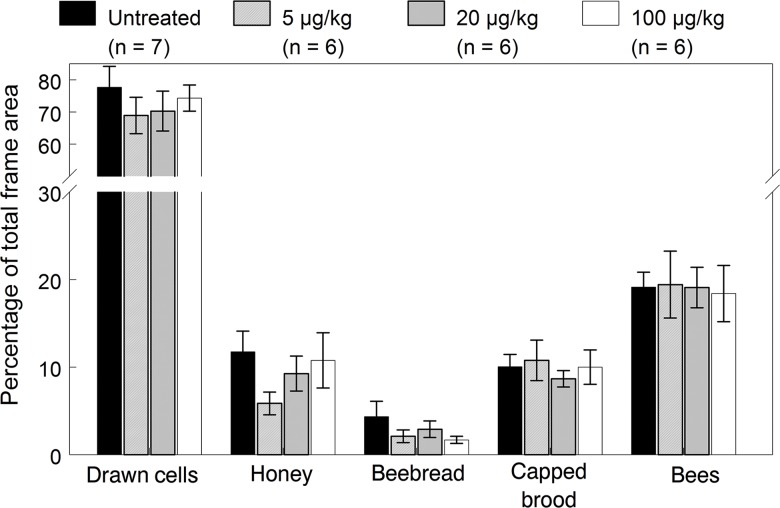
Colony performance endpoints (mean±SE) of the 2010 colonies recorded on October 7 prior to overwintering (about seven weeks after exposure).

All colonies survived to the last inspection (October 7), except for one replicate in each exposed group. These colonies died out during September due to a lack of brood and virtually no stored food, despite the fact that each hive was provisioned with sucrose syrup since mid-August. Of the remaining colonies going into the winter, results showed no significant differences in colony size, brood or food stores among treatment groups at the last inspection (Fig. 7, S4 Table). However, several colonies in the control and exposed groups contained less than the 6 kg of stored honey going into the winter. Overall winter survival was much lower than the levels experienced in 2009 as a result of over-consumption of food stores due to a mild winter. On February 7, hive inspections revealed 2, 2, 3 and 3 dead colonies in the control, 5, 20 and 100 μg/kg treatment groups, respectively, and most of these colonies were low in food stores and had to be provisioned with bee candy. A final inspection on March 17 confirmed another dead colony in each of the control and 5 μg/kg treatment groups. Of the 12 colonies that died out during the winter, only two hives lacked stored food which most likely led to starvation. Out of seven replicate colonies, 4, 3, 3 and 3 in the control, 5, 20 and 100 μg/kg groups, respectively, were able to successfully overwinter; however, Fisher’s one-tailed Exact Test showed no statistically significant (*p* = 0.21) in the proportions of overwintered colonies. After the March inspection, all remaining colonies were fed sucrose syrup in top feeders and were able to buildup normally in the spring.

#### Foraging measurements

**Fig 8:**
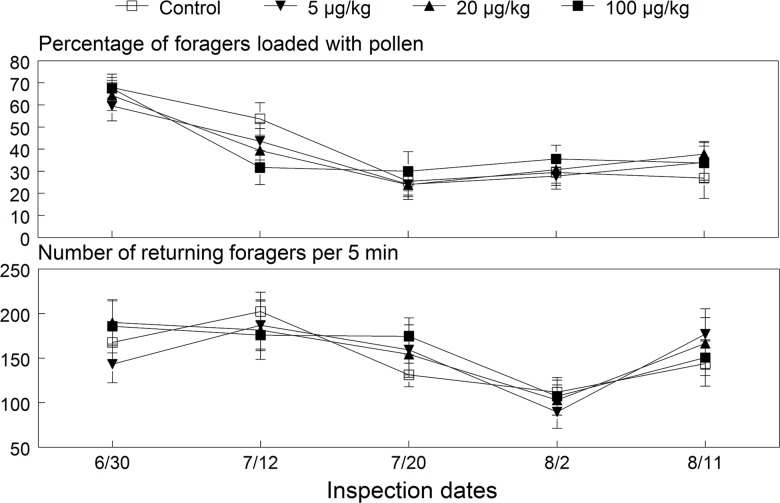
Mean (±SE) percentage of foragers with pollen pellets and the number of foragers returning to the 2010 colonies exposed to untreated or imidacloprid-spiked diet patties. ANOVA results for the percentage loaded with pollen (dose: *F*_(3, 42.6)_ = 0.85, *p* = 0.473; date: *F*_(4,81.7)_ = 18.93, *p* < 0.001; interaction: *F*_(12,82.2)_ = 0.93, *p* = 0.520) and number of foragers (dose: *F*_(3,34)_ = 1.13, *p* = 0.350; date: *F*_(4,80.8)_ = 8.64, *p* < 0.001; interaction: *F*_(12,80.9)_ = 0.71, *p* = 0.742).

The amount of pollen collected at the entrance of colonies varied widely over the season in response to changes in foraging sources but was not significantly different among treatment groups (*F*_(3,24.1)_ = 0.41, *p* = 0.75). Regression results also showed no significant relationship between the cumulative AUC values for collected pollen and imidacloprid dose (*p* = 0.88). Control, 5, 20 and 100 μg/kg exposed colonies collected an overall average (±SE) of 29.8±2.26, 35.1±2.93, 39.4±3.74 and 34.1±2.97 g of pollen per day, respectively. These levels were 41% less than the weight of pollen collected by the 2009 colonies, which had 31% more forager bees on average. Control colonies collected significantly less pollen during July due to breaks in brood rearing and reduced bee strength. Foraging activity recorded five times during the exposure period showed no evidence of any dose or interaction effects on the total number of foragers returning or the percentage of bees loaded with pollen pellets (Fig. 8). Overall, an average of 151 to 159 foragers returned per 5 min and 38 to 40% were loaded with pollen.

### 2011 Within-hive Fate Experiment

This study provided information on the actual within-hive residues of imidacloprid that was relevant to exposure doses used in the sublethal experiments, particularly residues in bees and brood resulting from contaminated diet patties. Table 1 summarizes the frequency of positive detections and range of imidacloprid residues found in various hive matrices at different collection times. The majority of the 87 samples from the control colonies had non-detectable residues, although eight showed trace levels that were apparently due to drifting or possible cross-contamination during sampling. For unexplained reasons, one bee sample collected after 2 weeks of exposure from an untreated colony contained 2.9 μg/kg and was analyzed twice for confirmation. Since this value lies more than 1.5 times outside the interquartile range of the residue data, it was considered an outlier and possibly due to a mislabeled sample. The frequency of positive detections and residue levels were significantly higher in colonies fed 100 μg/kg diet patties compared to residues in colonies fed 20 μg/kg sucrose syrup. Totaled over all hive matrices, 77.1 and 19.4% of the samples collected during exposure from colonies fed treated diet patties and sucrose syrup, respectively, contained residues of imidacloprid. The highest levels were found in honey (average 6.5–7.2 μg/kg) collected from colonies exposed to treated diet patties, followed by lower but consistent levels in bees (average 0.3–0.7 μg/kg) and beebread (average 0.9–1.0 μg/kg). Residues in larvae were non-detectable, except for one sample collected after 2 weeks of exposure in each treatment group. At 6 weeks after exposure, residue levels were slightly lower but the frequency of positive samples remained about the same in bees, beebread and honey from colonies fed diet patties with 100 μg/kg of imidacloprid. In contrast, residues in colonies fed 20 μg/kg in sucrose syrup declined more quickly after exposure. All samples of royal jelly from colonies fed 100 μg/kg diet patties had detectable levels of imidacloprid residues, ranging from 0.3 to 1.0 μg/kg (average 0.6 μg/kg), whereas no royal jelly samples from colonies fed treated sucrose syrup contained detectable levels. Residues of the imidacloprid metabolites (olefin, 5-hydroxy, urea, desnitro olefin, and chloronicotinic acid) were not detected in any of the samples. However, the LOD levels were higher (ranging from 0.4 to 4.4 μg/kg) for these degradates.

**Table 1:** Residues of imidacloprid in hive matrices resulting from feeding spiked Mega-bee diet patties and sucrose syrup to honey bee colonies for six weeks.

Treatment^a^	Sample duration	Number of colonies with positive detections/total samples (Range of imidacloprid residues^b^ (μg/kg) in positive samples)				
		Bees	Beebread	Larvae	Honey	Royal jelly
Colonies fed diet patties (100 μg/kg imidacloprid) and untreated sucrose syrup	2 weeks exposure	6 / 8 (0.2–1.4)	8 / 8 (0.5–1.7)	1 / 8 (0.4)	7 / 8 (4.7–13.4)	NA
	6 weeks exposure	5 / 8 (0.5–1.9)	8 / 8 (0.6–1.2)	0 / 8 (ND)	7 / 8 (2.8–10.8)	8 / 8 (0.3–1.0)
	6 weeks after exposure	8 / 8 (0.3–0.5)	8 / 8 (0.8–1.4)	0 / 7 (ND)	8 / 8 (2.3–11.7)	NA
Colonies fed sucrose syrup (20 μg/kg imidacloprid) and untreated diet patties	2 weeks exposure	3 / 8 (0.2–0.5)	2 / 8 (0.7–1.0)	1 / 8 (0.5)	4 / 8 (0.2–3.7)	NA
	6 weeks exposure	0 / 8 (ND)	3 / 8 (0.2–0.9)	0 / 8 (ND)	1 / 8 (0.9)	0 / 8 (NA)
	6 weeks after exposure	0 / 8 (ND)	2 / 8 (0.2–0.3)	0 / 5 (ND)	2 / 8 (0.2–0.5)	NA
Colonies fed untreated diet patties and sucrose syrup	2 weeks exposure	4 / 8 (0.2–2.9)	1 / 8 (1.1)	0 / 6 (ND)	0 / 8 (ND)	NA
	6 weeks exposure	0 / 8 (ND)	0 / 8 (ND)	0 / 6 (ND)	0 / 8 (ND)	0 / 6 (ND)
	6 weeks after exposure	0 / 8 (ND)	0 / 8 (ND)	0 / 4 (ND)	3 / 7 (0.2–0.3)	NA

^a^All colonies were fed 2000 g of sucrose syrup and 400 g of diet patties weekly for six weeks; thus each colony was exposed to 40 μg of imidacloprid per week.

^b^LOD = 0.2 μg/kg; LOQ = 0.6 μg/kg; ND = non-detectable; NA = not sampled.

Overall colony performance of the three treatment groups provided additional data on the sublethal effects of imidacloprid. Colony size was not significantly affected by the two exposure routes during the 6-week period (*F*_(2, 28.6)_ = 0.63, *p* = 0.54). Hive populations averaged 14,001±476, 13,890±408 and 13,080±338 bees per colony in treatment groups exposed to untreated food, 20 μg/kg sucrose syrup, and 100 μg/kg diet patties, respectively. However, bee strength measured during August inspections showed significant differences in the number frames with bees (*F*_(2, 14)_ = 8.44, *p* = 0.004). Colonies fed 100 μg/kg diet patties had 14–26% fewer frames of bees than the control colonies or colonies fed 20 μg/kg sucrose syrup. All other endpoints including brood rearing and food stores were not significantly affected by the exposure routes. All eight colonies in each treatment group were active on September 24 but 3, 4 and 4 colonies in the control, 20 μg/kg sucrose syrup, and 100 μg/kg diet patty groups, respectively, were rated weak with very little brood and stored food. Nearly an equal number of colonies in each group were rated either medium strength or strong. A contingency table *X* ^2^ analysis indicated that the proportions in each rating were independent of the treatment group (*p* = 0.98). After the last inspection, weak colonies were combined with stronger ones for overwintering and no further data were recorded.

## Discussion

### 

#### Imidacloprid exposure and hive residues

We designed the sublethal experiments to directly expose bees to known doses of imidacloprid as the only stressor and to force colonies to consume a significant portion of their protein requirement from the Mega-bee diet patties. MegaBee protein supplement is readily consumed by bees at about the same rate and closely resembles the nutritional value of pollen [66,67]. The patties contained approximately 16% protein and 37% carbohydrates based on the ratio of water, sucrose and Mega-bee powder. The daily consumption rates of diet patties (30–60 g) provided enough protein to support the daily development of up to 1260 larvae [68] or 1477 nurse bees [69]. In the same way, the consumed amount of sugar in patties was enough to support the daily development of up to 376 larvae, 70 brood bees, or 246 forager bees [69]. Though we cannot equate MegaBee diet consumption directly to bee-collected pollen or nectar, diet patty consumption alone provided a significant portion of the daily nutritional requirements of the colonies.

The presence of imidacloprid in bees and beebread collected weeks after exposure provided evidence that colonies were exposed for at least two or possibly three development cycles of brood. Residue levels were generally higher in beebread than in bees and more consistent in samples from colonies exposed to the higher treatment doses. Average residue levels of the positive detections ranged up to 3.7 μg/kg, and the majority of bee and beebread residues exceeded concentrations of imidacloprid found in bee-collected pollen, honey and bees reported from colony surveys [41–43,50]. It also is noted that traces of imidacloprid were detected in a few samples from control colonies in 2009, and this cross-contamination was apparently due to drifting and possibly some robbing because hives were placed close to each other in apiaries. Averaged across all negative and positive samples, these residues were well below levels found in treated colonies, were probably introduced into control colonies after the exposure period, and were not detected in the 2010 sublethal study. Nevertheless, this contamination underscores the importance of closing out declining colonies before robbing occurs, and taking the necessary steps to avoid contamination while sampling.

The within-hive fate experiment provided insights into the relative levels and distribution of imidacloprid residues among hive matrices resulting from doses used in the sublethal experiments. Colonies that were fed diet patties spiked with 100 μg/kg for six weeks showed residue levels in bees, beebread and honey that were close to or exceeded those dietary doses of imidacloprid that caused sublethal effects in the laboratory [38]. In particular, nearly all honey samples had detectable imidacloprid residues ranging from 2.3–13.4 μg/kg, even six weeks after exposure and more than 10 times higher than residues in bees and beebread. Residues were probably concentrated by the evaporative processing of nectar. Surprisingly, we expected higher residue levels in honey from colonies fed treated sucrose syrup, which exposed bees to the same quantity of imidacloprid each week. This suggests that hive bees were utilizing diet patties differently from the way bee-collected pollen is processed. It is generally agreed that pollen diet supplements are processed for food quickly and not stored for long periods of time. Laboratory feeding studies using marker dyes in diet patties have also demonstrated rapid deposition of the dye in bees [70]. The residue levels in honey were several times higher than the reported concentrations of imidacloprid in pollen collected from seed-treated sunflower, maize and canola [39,41–43] and even higher than residues found in beebread, honey and wax samples collected from colonies [50,21]. Imidacloprid residues in bees dropped to lower or non-detectable levels and were relatively absent in larvae at six weeks after exposure, whereas levels in beebread and honey remained relatively stable. The residue concentration in bees and larvae was probably reduced by the rapid metabolism of imidacloprid, which has a reported half-life of 4–5 hours in the honey bee [71,28], and further diluted by the addition of bee-collected uncontaminated nectar and pollen entering the colonies. In view of the glandular secretion and processing involved in producing royal jelly, we were surprised that all samples of royal jelly tested positive for imidacloprid residues. Residues in stored honey, larvae and royal jelly were not analyzed in the sublethal experiments; however, all life stages in the exposed colonies may have been subjected to higher levels of imidacloprid than the levels detected in beebread, assuming the same relative distribution of residues in hive matrices shown in the within-hive fate experiment. Based on the overall residue results, we would argue that the high dose of 100 μg/kg exposed continuously in diet patties for 12 weeks represented a worst-case scenario of field exposure to imidacloprid at the whole colony level.

#### Effects on foraging activity

Several laboratory and semi-field studies have shown that the sublethal effects of imidacloprid can interfere with food collection by affecting the longevity, olfactory learning and orientation functions of foraging bees (reviews in [38,58]). However, these effects have not been demonstrated at the colony level under relevant field exposure conditions. In our studies, we assumed that exposure to contaminated nectar or water while foraging was minimal because apiaries were isolated from any bee-attractive crops that may have been treated with imidacloprid. More likely, foragers returning to the treated colonies were either exposed to imidacloprid residues by consuming honey in the colony, or exposed earlier in their life as larvae fed brood food or as young nurse bees consuming pollen. This cumulative exposure could lead to subtle adverse effects on longevity, learning ability and homing behavior of forager bees. However, we found no evidence that imidacloprid affected foraging activity during and after exposure in both sublethal experiments. The number of foragers returning and percentage loaded with pollen pellets changed significantly over time in response to seasonal pollen sources but neither treatment nor its interaction with time had a significant effect on these endpoints. The weight of bee-collected pollen trapped at the hive entrance was considered a direct measure of foraging success. There were no significant differences in pollen weights among treatment groups in both sublethal experiments, except for the 2010 control colonies that collected less pollen during July than treated colonies due to lower bee populations. Interestingly, the overall amount of pollen trapped approximated closely the weight of diet patties consumed per day by each colony. Hive bees apparently were forced to utilize the diet patties for nutrition in place of pollen removed by the entrance traps. This further indicates that bees and brood were exposed to relatively high dietary concentrations of imidacloprid.

Other field studies have examined foraging behavior of bees fed sublethal doses of imidacloprid in sucrose water to represent exposure via contaminated nectar. Schneider et al. [72] fed individual foragers spiked sucrose solutions and recorded no effect on foraging behavior at imidacloprid doses of 1.9 μg/kg by bee weight but less foraging activity and longer foraging flights at 10x higher doses. Bortolotti et al. [73] showed no effects on the number of returning foragers but delays in return flights to feeding sites when bees were fed 100 μg/kg in sucrose solutions. A recent study by Feltham et al. [74] demonstrated that foraging efficiency of bumble bees dropped 31% when pollen was contaminated with sublethal levels (6 μg/kg) of imidacloprid but no effect on nectar foraging when fed syrup at 0.7 μg/kg. It is difficult to directly relate these results to those of our experiments because individual bees were exposed to single doses and different endpoints were measured. In our studies, imidacloprid concentrations in bees reached average levels up to 3.7 μg/kg and 2.8 μg/kg, respectively, from colonies exposed to 20 and 100 μg/kg doses. Considering the rapid metabolism of imidacloprid by honey bees, foragers were probably subjected to a chronic dose exposure of imidacloprid well exceeding levels that might be encountered by feeding on nectar in imidacloprid seed-treated fields. Taken altogether, these results provide evidence that continuous exposure of field relevant imidacloprid doses had no significant effects on foraging activity at the colony level.

#### Effects on colony health

The presence of an active egg laying queen in pheromonal control of colony integrity, sufficient ratio of bees to brood to maintain population growth, relatively disease/pest free, and adequate nutrition are principal determinants of a healthy honey bee colony. Sublethal exposure to imidacloprid can affect these factors in many ways. In this study, we monitored the performance of each colony by detailed assessments of bee population, brood (capped cells and older larvae), beebread and stored honey during and after exposure until the fall. We also recorded the percentage area of foundation with fully drawn cells as an indicator of colony strength. In both years, development of the bee population and brood rearing were not affected by the exposure doses. At the end of exposure in 2009, colonies among treatment groups ranged in size from 21,298 to 24,160 bees and had ratios of 1.4 to 1.7 adult bees to one capped brood cell. Ratios increased to nearly 3 in early October as brood rearing declined and colonies prepared for overwintering. Colonies in 2010 were smaller in size ranging from 13,393 to 15,435 bees at the end of exposure, with ratios of 1.1 to 2.3 adult bees to capped brood. The percentages of frame area with beebread, capped honey and drawn cells showed no statistically significant differences due to imidacloprid doses in both years. However, overall dose relationship trends showed some evidence of an imidacloprid effect depending on the study year. For example, the dose relationship with honey stores in 2009 using regression analysis was significant, indicating higher stores with increasing doses of imidacloprid and suggesting that exposed colonies may have avoided the contaminated food. Based on results in Table 1, the honey stores likely contained the highest concentrations of the insecticide. Bee population and the portion of drawn cells were higher with increased dose in 2009, and beebread stores decreased with dose in 2010. However, these regression relationships were not significant.

The presence of diseases and pest species was not considered a significant stress factor affecting colony health. Infection rates of parasitic mites and *Nosema* spores were low and normal for first year packaged colonies. However, *Varroa* infestations did show a significant dose response in both years, and the 100 μg/kg treated colonies in 2009 had statistically higher mite counts compared to counts in control colonies. This effect could possibly have greater impacts on colony health if colonies are sublethally exposed to imidacloprid over multiple years, particularly since exposure to neonicotinoids can compromise immune defense responses in honeybees [75]. Despite these dose-related patterns, the general health of colonies based on all endpoints taken together surprisingly showed no measureable differences among treatment groups during or shortly after the exposure period. However, results did show evidence of delayed sublethal effects later in September, when several colonies exposed to the higher doses of imidacloprid in 2009 became weak due to higher rates of queen loss and broodless periods. These colonies could not recover after queen supersedure and either died out or were terminated to avoid robbing. One colony in each exposed group in 2010 also died out during late September due to lack of brood and stored food, despite being fed supplemental sucrose syrup. Of the 2010 colonies that did survived to October, most imidacloprid-exposed colonies had numerically fewer bees and less brood, beebread and honey going into the winter. Pooled over both years, 100, 94.1, 82.4 and 82.4% of the colonies in the control, 5, 20 and 100 μg/kg treatment groups, respectively, survived to the last inspection in October. Although not statistically significant, this overall dose-dependent response strongly suggests that the higher imidacloprid doses had delayed sublethal effects on colony health. Time-to-effect studies have shown that the effects of chronic exposure to relatively low doses of imidacloprid can accumulate in insects, resulting in delayed toxicity [76].

#### Effects on overwintering

Requirements for successful overwintering of a honey bee colony include an adequate population to maintain cluster temperature, proper age of bees going into the winter, and sufficient stores of honey and pollen. Each colony in 2009 and 2010 was fed supplemental sucrose syrup during the fall to provision at least 30 kg of stored honey. However, many 2010 colonies in all treatment groups inspected in February were low in food stores and had to be provisioned with bee candy. In both years, the proportion of colonies with a queen and active brood nest observed in March was considered an indication of successful overwintering. Results of the 2009 experiment suggested that chronic sublethal exposure to imidacloprid during the summer weakened colonies and affected their ability to overwinter. Average survival rates in control, 5, 20 and 100 μg/kg treatment groups in March were 100, 80, 70 and 60%, respectively. Relatively fewer colonies from the 2010 experiment survived the winter, with only 57% of the control colonies survived compared to an overall 43% of the exposed colonies. We contribute this higher mortality to subnormal colonies going into the winter and abnormally higher temperatures during the winter which resulted in over-consumption of the stored food. Additionally, the disproportionate queen losses early in the season and subsequent reduced performance of the 2010 control colonies may have prejudiced the statistical analysis of treatment effects. None of the dead-out colonies in both experiments showed evidence of starvation or symptoms of colony collapse disorder, except several colonies in 2010 completely lacked food and likely died from starvation.

**Fig 9:**
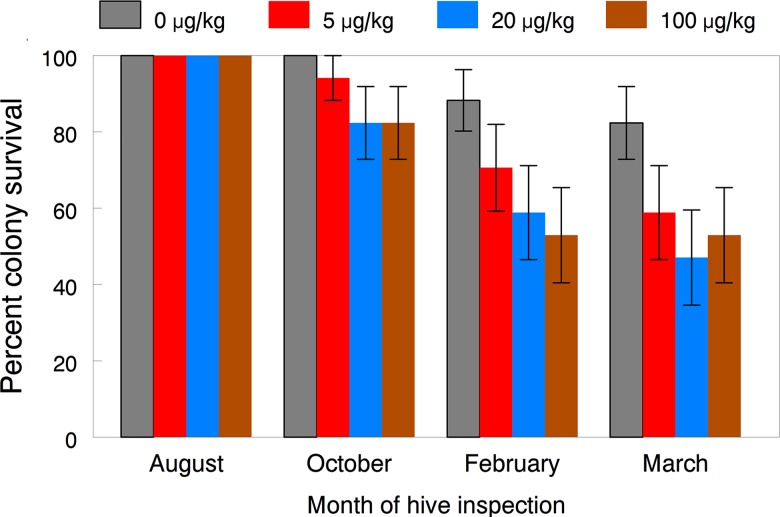
Proportion of colonies exposed for 12 weeks to untreated or imidacloprid-spiked diet patties and survived to August (end of exposure), October, February and March (successful overwintering). Means (±SE) pooled over both years are given for each exposure group and inspection month. Logistic regression results for the overall dose effect (*F*_(3, 15)_ = 4.21, *p* = 0.024; significant of differences between control colonies and colonies exposed to 5 μg/kg (*p* = 0.201), colonies exposed to 20 μg/kg (*p* = 0.027), and colonies exposed to 100 μg/kg (*p* = 0.027).

Trends in colony survival with higher levels of imidacloprid exposure varied widely between years. Statistical analyses of the proportion of colonies surviving each year showed no differences in 2010 and only the proportion surviving in the 100 μg/kg treated group was significantly lower than that of the control colonies in 2009. However, statistical power was increased by pooling the colony survival data over the October, February and March inspections of both years. The logistic regression results of the combined data showed an overall significant dose effect (*F*_(3,15)_ = 4.21, *p* = 0.024), and all contrast tests comparing survival between control and each treatment group were also significant, except for colonies exposed to 5 μg/kg. Pooled over both years, colony survival in March averaged 82.4, 58.8, 47.1 and 52.9% in the control, 5, 20 and 100 μg/kg treatment groups, respectively (Fig. 9). These results, along with dose-response patterns for several colony performance endpoints prior to overwintering, clearly indicate negative impacts on honey bee colony health due to the cumulative sublethal exposure to 20 and 100 μg/kg of imidacloprid for 12 weeks during the early summer. The delayed effect of reduced winter survival was apparently due to higher rates of queen loss and broodless periods during the late summer. Interestingly, the 2009 colonies entering the winter were seemingly stronger than the 2010 colonies, yet they experienced winter mortality that was more positively associated with the level of imidacloprid exposure. We contribute this to the queenless situation that probably resulted in a higher than normal proportion of old bees going into the winter. When this occurs, although colony size may appear adequate in the fall, the bee cluster decreases faster than brood rearing can compensate in February and March and the colony can fail. The study by Sandrock et al. [63] also reported that queen failure significantly contributed to colony weakening but not overwintering loss in colonies exposed over 1.5 months to pollen patties contaminated with thiamethoxam and clothianidin at worst-case exposure scenarios for seed-treated crops. Exposure doses in this study were comparable to our lowest dose of 5 μg/kg of imidacloprid, which had no short-term effects on colony performance.

### Conclusions

To our knowledge, this study is the first to examine the chronic sublethal effects on whole honey bee colonies subjected to worse-case scenarios as well as normal dietary exposure (5μg/kg) to imidacloprid. We used spiked diet patties placed within colonies to deliver continuous direct exposure over multiple brood cycles to imidacloprid residues that were generally higher than levels found in bee-collected pollen and nectar under field conditions. Our results provide evidence that imidacloprid exposure doses up to 100 μg/kg had no significant effects on foraging activity or colony performance during and shortly after 12 weeks of exposure. However, several colony performance endpoints showed dose-response patterns, particularly higher *Varroa* infestations with increased dose, though not all patterns were statistically significant. The major finding was the higher rates of queen replacement and resulting broodless periods during the late summer in colonies exposed to 20 and 100 μg/kg of imidacloprid, which led to weaker colonies going into the winter. These exposure regimes sublethally affected colony health and significantly reduced overwintering success. However, the question remains as to whether doses of 100 or even 20 μg/kg exposed for 12 continuous weeks realistically represent imidacloprid residues in bee-collected food under agriculture settings. In certain field situations, residues of imidacloprid can reach or exceed 100 μg/kg in pollen of treated crops during several weeks of flowering [35] or in guttation droplets exuded from treated corn seedlings [45,46]. However, it is uncommon for honey bees to be exposed to these doses for extended periods. Furthermore, bees generally forage on different water and floral sources simultaneously and not all sources will contain residues; thus their foraging behavior tends to reduce the concentration of imidacloprid in food stored in the colony. The within-hive fate experiment demonstrated that imidacloprid residues of 100 μg/kg in diet patties or 20 μg/kg in sucrose syrup became diluted or non-detectable due to the processing of beebread and honey and the rapid metabolism of the chemical by bees. Given the weight of evidence presented here, we conclude that chronic exposure to imidacloprid at the higher range of field doses (20 to 100 μg/kg) in the pollen of certain treated crops could contribute to reduced overwintering success but the most likely encountered field doses of 5 μg/kg, especially relevant for seed-treated crops, have negligible effects on honey bee colony health. Currently there is wide agreement that sublethal exposure to imidacloprid can cause adverse effects on honey bees in laboratory studies [77] but no evidence that this widely used insecticide is the major stressor causing colony declines. Our findings agree with a causal analysis by Staveley et al. [37] that judged neonicotinoid pesticides to be an unlikely sole cause of colony declines. Finally, this study makes evident the importance of conducting risk assessment studies on honey bee colonies over longer periods to reveal the chronic sublethal effects on queen health and bee behaviors that can ultimately impair colony performance [78].

## Supporting Information

S1 Table Effects of imidacloprid doses on the performance of the 2009 colonies exposed to untreated or spiked diet patties for 12 weeks. Data were collected on August 6 at the end of the exposure period.
(PDF)

S2 Table Effects of imidacloprid doses on the performance of the 2009 colonies exposed to untreated or spiked diet patties for 12 weeks. Data were collected on October 6 prior to overwinter and two months after exposure.
(PDF)

S3 Table Effects of imidacloprid doses on the performance of the 2010 colonies exposed to untreated or spiked diet patties for 12 weeks. Data were collected on August 19 at the end of the exposure period.
(PDF)

S4 Table Effects of imidacloprid doses on the performance of the 2010 colonies exposed to untreated or spiked diet patties for 12 weeks. Data were collected on October 7 prior to overwinter and about seven weeks after exposure.
(PDF)
