# Correction: Assessment of Chronic Sublethal Effects of Imidacloprid on Honey Bee Colony Health

**DOI:** 10.1371/journal.pone.0126043

**Published:** 2015-04-24

**Authors:** Galen P. Dively, Michael S. Embrey, Alaa Kamel, David J. Hawthorne, Jeffery S. Pettis

In the Results section, there is an error in the third sentence of the first paragraph where the units of imidacloprid are incorrectly stated in mg instead of μg. The correct sentence is: Based on total consumption over 12 weeks, each colony of the 5, 20 and 100 μg/kg treatment groups was exposed to an average cumulative dose of 16.6, 63.7 and 322.6 μg of imidacloprid, respectively.
