# Correction: Assessment of Chronic Sublethal Effects of Imidacloprid on Honey Bee Colony Health

**DOI:** 10.1371/journal.pone.0181297

**Published:** 2017-07-07

**Authors:** Galen P. Dively, Michael S. Embrey, Alaa Kamel, David J. Hawthorne, Jeffery S. Pettis

The following information is missing from the Competing Interest statement: Although this research was not financially supported by organizations that may gain or lose financially through its publication, one of the author (GPD) has received financial support for other research from Syngenta Seeds and Pioneer/DuPont. This does not alter our adherence to PLOS ONE policies on sharing data and materials.
