# Supplementary material for: Assessment of Chronic Sublethal Effects of Imidacloprid on Honey Bee Colony Health
Source: PLoS One. 2015 Mar 18;10(3):e0118748. doi: 10.1371/journal.pone.0118748 (PMC4364903; doi:10.1371/journal.pone.0118748)
Supplement: S1 Table — Data were collected on August 6 at the end of the exposure period. (PDF) [file pone.0118748.s001.pdf]

Table S1. Effects of imidacloprid doses on the performance of the 2009 colonies exposed to untreated or spiked diet patties for 12 weeks. Data were collected on August 6 at the end of the exposure period.

	Exposure dose<br>Mean (± SE) percentage of total frame area covered				ANOVA results		
Performance endpoint	Untreated	5 µg/kg	20 µg/kg	100 µg/kg	Df	F	*p* value
Bees	27.6±3.13	31.5±2.75	26.9±3.32	28.5±2.88	3, 31	0.68	0.571
Capped brood	9.2±1.13	8.9±1.21	8.12±1.68	8.12±1.43	3, 31	0.28	0.841
Capped honey	11.7±2.39	13.1±2.71	13.1±2.33	19.3±2.47	3, 31	1.82	0.165
Beebread	5.7±1.16	7.2±1.64	7.6±1.67	8.00±1.28	3, 35	0.46	0.712
Drawn out cells	59.5±5.38	66.8±5.17	61.1±4.82	70.3±3.53	3, 31	1.04	0.390
