# Supplementary material for: Assessment of Chronic Sublethal Effects of Imidacloprid on Honey Bee Colony Health
Source: PLoS One. 2015 Mar 18;10(3):e0118748. doi: 10.1371/journal.pone.0118748 (PMC4364903; doi:10.1371/journal.pone.0118748)
Supplement: S2 Table — Data were collected on October 6 prior to overwinter and two months after exposure. (PDF) [file pone.0118748.s002.pdf]

Table S2. Effects of imidacloprid doses on the performance of the 2009 colonies exposed to untreated or spiked diet patties for 12 weeks. Data were collected on October 6 prior to overwinter and two months after exposure.

	Exposure dose<br>Mean (± SE) percentage of total frame area covered				ANOVA results		
Performance endpoint	Untreated	5 µg/kg	20 µg/kg	100 µg/kg	Df	F	*p* value
Bees	24.5±1.48	21.4±2.10	20.9±2.70	23.1±1.85	3, 33	1.35	0.254
Capped brood	4.01±0.51	3.7±0.76	3.9±0.77	4.4±0.66	3, 33	0.00	0.990
Capped honey	22.8±3.28	21.9±2.85	24.8±2.81	27.6±2.47	3, 33	0.69	0.556
Beebread	2.4±0.64	4.0±0.72	3.7±0.94	3.3±0.57	3, 33	0.95	0.428
Drawn out cells	65.3±4.53	71.6±3.53	68.2±3.71	75.2±2.18	3, 33	1.29	0.295
