# Supplementary material for: Assessment of Chronic Sublethal Effects of Imidacloprid on Honey Bee Colony Health
Source: PLoS One. 2015 Mar 18;10(3):e0118748. doi: 10.1371/journal.pone.0118748 (PMC4364903; doi:10.1371/journal.pone.0118748)
Supplement: S3 Table — Data were collected on August 19 at the end of the exposure period. (PDF) [file pone.0118748.s003.pdf]

Table S3. Effects of imidacloprid doses on the performance of the 2010 colonies exposed to untreated or spiked diet patties for 12 weeks. Data were collected on August 19 at the end of the exposure period.

	Exposure dose<br>Mean (± SE) percentage of total frame area covered				ANOVA results		
Performance endpoint	Untreated	5 µg/kg	20 µg/kg	100 µg/kg	Df	F	*p* value
Bees	23.6±2.41	19.9±2.98	19.5±2.37	21.1±1.81	3, 24	0.59	0.623
Capped brood	6.7±1.51	8.5±2.35	9.05±2.18	10.1±1.60	3, 18	1.01	0.412
Capped honey	3.9±1.54	2.3±0.70	2.2±0.119	8.8±2.82	3, 24	1.34	0.286
Beebread	9.5±2.68	6.7±2.46	5.9±1.73	6.2±0.79	3, 18	0.65	0.595
Drawn out cells	73.3±7.03	64.5±5.24	66.4±6.36	70.1±4.51	3, 24	0.45	0.722
