# Supplementary material for: Assessment of Chronic Sublethal Effects of Imidacloprid on Honey Bee Colony Health
Source: PLoS One. 2015 Mar 18;10(3):e0118748. doi: 10.1371/journal.pone.0118748 (PMC4364903; doi:10.1371/journal.pone.0118748)
Supplement: S4 Table — Data were collected on October 7 prior to overwinter and about seven weeks after exposure. (PDF) [file pone.0118748.s004.pdf]

Table S4. Effects of imidacloprid doses on the performance of the 2010 colonies exposed to untreated or spiked diet patties for 12 weeks. Data were collected on October 7 prior to overwinter and about seven weeks after exposure.

	Exposure dose<br>Mean (± SE) percentage of total frame area covered				ANOVA results		
Performance endpoint	Untreated	5 µg/kg	20 µg/kg	100 µg/kg	Df	F	*p* value
Bees	19.1±1.72	19.4±3.82	19.1±2.31	18.4±3.22	3, 18	0.20	0.896
Capped brood	10.0±1.41	10.8±2.32	8.7±0.94	10.0±1.97	3, 24	0.32	0.810
Capped honey	11.7±2.36	5.85±1.29	9.25±2.00	10.76±3.16	3, 24	1.45	0.253
Beebread	4.4±1.75	2.1±0.73	2.9±0.94	1.7±0.41	3, 18	1.18	0.345
Drawn out cells	77.7±6.49	68.9±5.65	70.3±6.19	74.3±4.07	3, 18	1.00	0.416
